# Opposite effects of acute and chronic IGF1 on rat dorsal root ganglion neuron excitability

**DOI:** 10.3389/fncel.2024.1391858

**Published:** 2024-06-11

**Authors:** Jennyfer Pastor, Bernard Attali

**Affiliations:** Department of Physiology and Pharmacology, Faculty of Medicine and Health Sciences and Sagol School of Neurosciences-Tel Aviv University, Tel Aviv, Israel

**Keywords:** IGF-1, dorsal root ganglion neuron, TRPV1, M-channel, IGF-1 receptor, potassium channel

## Abstract

Insulin-like growth factor-1 (IGF-1) is a polypeptide hormone with a ubiquitous distribution in numerous tissues and with various functions in both neuronal and non-neuronal cells. IGF-1 provides trophic support for many neurons of both the central and peripheral nervous systems. In the central nervous system (CNS), IGF-1R signaling regulates brain development, increases neuronal firing and modulates synaptic transmission. IGF-1 and IGF-IR are not only expressed in CNS neurons but also in sensory dorsal root ganglion (DRG) nociceptive neurons that convey pain signals. DRG nociceptive neurons express a variety of receptors and ion channels that are essential players of neuronal excitability, notably the ligand-gated cation channel TRPV1 and the voltage-gated M-type K^+^ channel, which, respectively, triggers and dampens sensory neuron excitability. Although many lines of evidence suggest that IGF-IR signaling contributes to pain sensitivity, its possible modulation of TRPV1 and M-type K^+^ channel remains largely unexplored. In this study, we examined the impact of IGF-1R signaling on DRG neuron excitability and its modulation of TRPV1 and M-type K^+^ channel activities in cultured rat DRG neurons. Acute application of IGF-1 to DRG neurons triggered hyper-excitability by inducing spontaneous firing or by increasing the frequency of spikes evoked by depolarizing current injection. These effects were prevented by the IGF-1R antagonist NVP-AEW541 and by the PI3Kinase blocker wortmannin. Surprisingly, acute exposure to IGF-1 profoundly inhibited both the TRPV1 current and the spike burst evoked by capsaicin. The Src kinase inhibitor PP2 potently depressed the capsaicin-evoked spike burst but did not alter the IGF-1 inhibition of the hyperexcitability triggered by capsaicin. Chronic IGF-1 treatment (24 h) reduced the spike firing evoked by depolarizing current injection and upregulated the M-current density. In contrast, chronic IGF-1 markedly increased the spike burst evoked by capsaicin. In all, our data suggest that IGF-1 exerts complex effects on DRG neuron excitability as revealed by its dual and opposite actions upon acute and chronic exposures.

## Introduction

Insulin-like growth factor 1 (IGF-1) is a 70–amino acid polypeptide hormone that acts as an endocrine growth factor in both paracrine and autocrine fashions (Yakar et al., 2005; Fernandez and Torres-Aleman, 2012). IGF-1 exhibits a ubiquitous distribution in various tissues and displays pleiotropic functions in both neuronal and non-neuronal cells (Hakuno and Takahashi, 2018). IGF-1 is primarily produced by the liver but can also be locally synthesized and released by neurons and astrocytes (Yakar et al., 2005; Quesada et al., 2007; Fernandez and Torres-Aleman, 2012). IGF-1 binds with high affinity to the IGF-1 receptor (IGF-1R) and with lower affinity to the insulin receptor (Hakuno and Takahashi, 2018). In the central nervous system (CNS), IGF-1R signaling regulates a variety of crucial functions including neurogenesis, axonal growth, brain development and aging, neural metabolism, as well as neuronal excitability, excitatory and inhibitory synaptic transmission or homeostatic plasticity (Nilsson et al., 1988; Castro-Alamancos and Torres-Aleman, 1993; Kenyon et al., 1993; D’Ercole et al., 1996; Seto et al., 2002; Holzenberger et al., 2003; Tropea et al., 2006; Kenyon, 2010; Fernandez and Torres-Aleman, 2012; Maya-Vetencourt et al., 2012; Gazit et al., 2016; Noriega-Prieto et al., 2021; Katsenelson et al., 2022). In addition to their presence in the CNS, IGF-1 and IGF-IR are also expressed in the peripheral nervous system, notably in the sensory dorsal root ganglion (DRG) nociceptive neurons that convey pain modalities. Many lines of evidence indicate that IGF1- and IGF-1R are expressed in small-diameter nociceptive DRGs and in spinal dorsal horn neurons that are closely related to pain signals (Craner et al., 2002; Hawkes and Kar, 2002; Dai et al., 2009; Takayama et al., 2011; Li et al., 2013; Wang et al., 2014; Zhang et al., 2014; Zhu et al., 2018; Tang et al., 2019; Ma et al., 2023).

DRG nociceptive neurons express a large panel of receptors and ion channels that are crucial players of neuronal excitability, notably the ligand-gated cation channel TRPV1 and the voltage-gated M-type K^+^ channel, which, respectively, triggers and reduces sensory neuron excitability (Caterina and Julius, 2001; Barkai et al., 2017; Du et al., 2018). The M-type K^+^ channel is encoded by the hetero-tetrameric assembly of the Kv7.2 or Kv7.5 and Kv7.3 subunits, which generate subthreshold, voltage-gated and non-inactivating K^+^ current also called M-currents (Marrion, 1997; Wang et al., 1998; Jentsch, 2000; Cooper and Jan, 2003; Delmas and Brown, 2005). Indeed, the M-currents are negatively modulated by muscarinic cholinergic receptor agonists (Brown and Adams, 1980; Halliwell and Adams, 1982; Marrion, 1997; Wang et al., 1998; Jentsch, 2000; Cooper and Jan, 2003; Delmas and Brown, 2005). The transient receptor potential vanilloid 1 (TRPV1) is a ligand-gated non-selective cation channel. TRPV1 activation causes an inward current that depolarizes the membrane potential up to a threshold that enables action potential generation and propagation along the sensory afferents of peripheral nociceptors (Caterina et al., 1997; Rosenbaum et al., 2022).

Many studies have found that the IGF-1/IGF-1R pathway can exert opposite actions on pain signals. Several works demonstrate that the IGF-1R signaling aggravates acute and chronic pain while others show that it is involved in relieving chronic pain (Ma et al., 2023). Although it is clear that the IGF-1R signaling tunes pain sensitivity, its modulation of two other important players of pain signals, the TRPV1 and M-type K^+^ channel remains unexplored yet. In this work, we examined the impact of IGF-1R signaling on cultured rat DRG neuron excitability and its modulation of TRPV1 and M-type K^+^ channel activities. Acute application of IGF-1 to DRG neurons produced hyper-excitability by inducing spontaneous firing or by increasing the frequency of spikes evoked by depolarizing current injection. These effects were prevented by the IGF-1R antagonist NVP-AEW541 and by the PI3Kinase blocker wortmannin. Unpredictably, acute exposure to IGF-1 profoundly inhibited both the TRPV1 current and the frequency of spikes evoked by capsaicin. While inhibition of the Src kinase by PP2 potently reduced the spike burst triggered by capsaicin, it did not affect the IGF-1 depression of capsaicin-evoked spikes. When DRG neurons were chronically treated with IGF-1 (24 h) the frequency of spikes evoked by depolarizing current injection was potently reduced and the M-current density was upregulated. In contrast, chronic IGF-1 treatment markedly increased the frequency of spikes evoked by capsaicin. Altogether, our data suggest that IGF-1 exerts complex effects on DRG neuron excitability as reflected by its dual and opposite actions following acute and chronic exposures.

## Materials and methods

### Animals

Neonatal (0–4 day-old) Wistar rats of either sex were used for generating the primary cultures of DRG neurons. All experimental protocols conformed to the guidelines of the Institutional Animal Care and Use Committee of Tel-Aviv University, Israel (authorization number 01-21-073), and to the guidelines of the NIH animal welfare.

### Drugs

Capsaicin and PP2 (Sigma-Aldrich, Rehovot, Israel), IGF-1 (Peprotech, Rehovot, Israel), XE991 and retigabine (RTG) (Alomone Labs, Jerusalem, Israel) were used in this study. XE991 was solubilized in double distilled water (DDW) for stock solution (10 mM). Capsaicin, PP2 and retigabine were solubilized in dimethyl sulfoxide (DMSO) for stock solutions (10 mM), stored at −20°C, and diluted with the recording solution at appropriate concentrations before the experiments to yield the final DMSO concentration of 0.01–0.1%. IGF-1 was initially reconstituted in DDW to a concentration of 1 mg/ml on ice, then diluted to a stock concentration of 10 μM in DDW containing 0.1% bovine serum albumin (BSA) and stored at −80°C until experiments to reach a final concentration of 100 nM.

### Primary rat DRG cultures

The number of animals and culture preparations used in these experiments was 86 (one neonatal rat per culture preparation). DRG neurons were dissected out from 0- to 4-day neonatal Wistar rats. DRGs were placed in DMEM and dissociated by enzymatic treatment. In brief, after 30-min incubation in 0.5 mg/ml trypsin type IX-S, 1 mg/ml collagenase type 1A, and 0.1 mg/ml DNase I (Sigma-Aldrich) in Ca2+ and Mg2+-free Dulbecco’s Modified Eagle Medium (DMEM), the ganglia were centrifuged and suspended in DMEM with 1.25 mg/ml trypsin inhibitor (Sigma-Aldrich). After 5-min incubation, the ganglia were again centrifuged, suspended in DMEM supplemented with 2 mM GlutaMAX, 10% fetal bovine serum, penicillin/streptomycin antibiotics and mechanically triturated with a fire-polished glass Pasteur pipette. For electrophysiological recording, dissociated DRG neurons were plated on 13-mm glass coverslips, previously coated with poly-L-lysine (0.5 mg/ml) and collagen (0.25 mg/ml), and used at 1-d *in vitro* culture.

### Electrophysiology

Patch clamp was performed in the whole-cell configuration. DRG neurons recordings were performed 1 day after dissection. Data were sampled at 10 kHz and low pass filtered at 4 kHz with Multiclamp 700B amplifier and pClamp10 software and a 4-pole Bessel low pass filter (Molecular Devices, Sunnyvale, CA, USA). The patch electrodes were pulled from borosilicate glass pipettes (Warner Instruments, Hamden, CT, USA) with a pipette resistance of 3–5 MΩ. For the current-clamp and voltage-clamp recordings, the patch pipettes were filled with the following internal solution: 135 mM KCl, 1 mM MgATP, 1 mM K_2_ATP, 2 mM EGTA (135 nM free Ca^2+^), 1.1 mM CaCl_2_, 5 mM glucose and 10 mM HEPES, adjusted with KOH to pH 7.3 (osmolarity was adjusted with sucrose to 290 mOsm). The external solution contained 145 mM NaCl, 2.5 mM KCl, 1.2 mM MgCl_2_, 5 mM glucose, 2 mM EGTA and 10 mM HEPES, adjusted with NaOH to pH 7.3 (osmolarity was adjusted with sucrose to 305 mOsm). Liquid junction potential was calculated (+4.5 mV) and subtracted from all recorded voltages. Series resistances ranged between 8 and 12 MΩ and were compensated for V-clamp recording using the adjust bottom Rs of the Multiclamp 700B amplifier (providing ≈85–90% compensation). For measuring the input resistance in the I-clamp configuration, small incremental negative currents (−20 to 150 pA) were injected to DRG neurons to construct an I-V linear relation, whose slope yielded the input resistance (Rin). The Rin values ranged between 550 and 650 MΩ. Input resistance, series resistance, baseline current, and capacitance were monitored throughout the experiments. Recordings were rejected if the series resistance changed by more than 20% during recording. All recordings with input resistance lower than 400 MΩ and series resistance larger than 20 MΩ, or unstable membrane capacitance were rejected. At −60 mV, baseline current ranged between 0 and −150 pA. The average cell capacitance (mean ± SEM) was 27.02 ± 1.47 pF (*n* = 287). 1 μM capsaicin exposure was evoked by rapid application using a fast perfusion system (AutoMate Scientific, Berkeley, CA, USA). For M-current recording, the internal solution contained 130 mM K-gluconate, 6 mM KCl, 2 mM K_2_ATP, 10 mM HEPES, 1.1 mM EGTA, 0.1 mM CaCl_2_ (10.9 nM free Ca^2+^), adjusted with KOH to pH 7.3 (osmolarity was adjusted with sucrose to 300 mOsm) and the extracellular solution contained 145 mM N-Methyl-D-glucamine hydrochloride, 2.5 mM KCl, 1.2 mM MgCl, 5 mM glucose, 10 mM HEPES, 2 mM EGTA, 1 mM 4-aminopyridine and 10 μM tetrodotoxin adjusted with HCl to pH 7.3 (osmolarity was adjusted with sucrose to 310 mOsm). To record the M-current, cells were held at −60 mV in the voltage-clamp configuration. A step to −20 mV was then given for 6 s to open the M-currents and remove residual inactivating voltage-dependent currents. Then, the voltage was brought back to −60 mV for 4 s to close M-currents, followed by another −20 mV step for 2 s. After offline leak subtraction, the M-current was calculated by subtracting the peak tail current at −60 mV in the presence of RTG (10 μM), to that in the presence of XE991 (10 μM).

### Data analysis

Data analysis was performed using the Clampfit program (pClamp10), Microsoft Excel (Microsoft, Richmond, WA, USA), and Prism 10.0 (GraphPad Software, Inc., San Diego, CA, USA). To analyze the afterhyperpolarization (AHP) size, we measured the AHP area after the first spike evoked either by depolarizing current injection or by capsaicin fast exposure. The AHP area was measured as the area under the baseline signal of the resting membrane potential immediately following the spike. Results were expressed as mean and standard error of the mean (SEM). In acute treatments that were carried out in the same neuron, statistical comparisons between two groups with Gaussian distributions were performed using a two-tailed paired *t*-test. For non-Gaussian distributions of the paired two groups, comparisons were performed using the non-parametric Wilcoxon test. For unpaired two groups, comparisons were performed using the non-parametric Mann–Whitney test. For more than 2 groups on the same neuron, comparisons were performed using a one-way ANOVA Kruskal–Wallis test. For chronic treatments involving two independent groups of cells with non-normal distribution, statistical comparisons were performed using non-parametric Mann–Whitney test, while those with normal distribution, comparisons were performed with a two-tailed unpaired *t*-test. For more than 2 groups, comparisons were performed using a one-way ANOVA Kruskal–Wallis test.

## Results

### Acute IGF-1 exposure increased intrinsic DRG neuron excitability but inhibited the TRPV1 current and the frequency of spikes evoked by capsaicin

**FIGURE 1:**
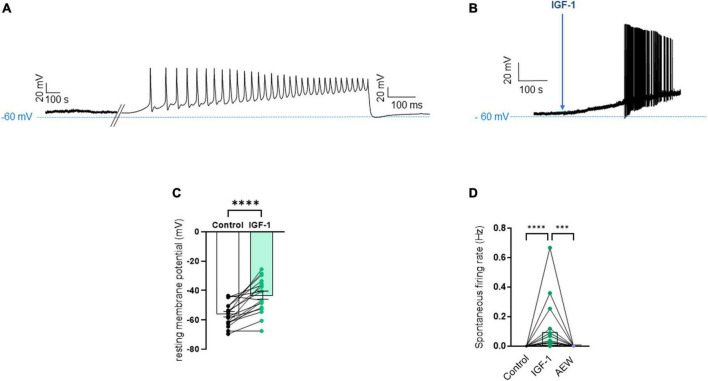
Acute exposure of DRG neurons to IGF-1 depolarizes the resting membrane potential and induces spontaneous activity. **(A)** Representative trace of the membrane potential a control DRG neuron that did not exhibit spontaneous firing but was excitable since it produced spikes upon a ramp of depolarizing current injection (from 0 to 600 pA for 800 ms). **(B)** Representative trace of the membrane potential a DRG neuron exposed to IGF-1 (100 nM) that induced depolarization and led within less than 2 min to spontaneous firing. **(C)** Statistics of the resting membrane potential: within 1 min application of 100 nM IGF-1, the resting membrane potential of DRG neurons significantly depolarized from −56.2 ± 1.8 mV to −43.5 ± 2.7 mV; *n* = 18; two-tailed paired *t*-test, *t* = 6.136, df = 17; ****P < 0.0001. **(D)** Statistics of spontaneous firing rate (Hz). Out of 18 recorded neurons, 12 exhibited spontaneous firing (Figure 1B), which was prevented by the prior incubation with the IGF-1R antagonist NVP-AEW541 (2 μM). The firing frequency (Hz) in the same neuron was 0, 0.094 and 0, in the absence, presence of IGF-1 (100 nM) and IGF-1+ NVP-AEW541, respectively; Kruskal–Wallis test, ****P < 0.0001 and ***P = 0.0002.

**FIGURE 2:**
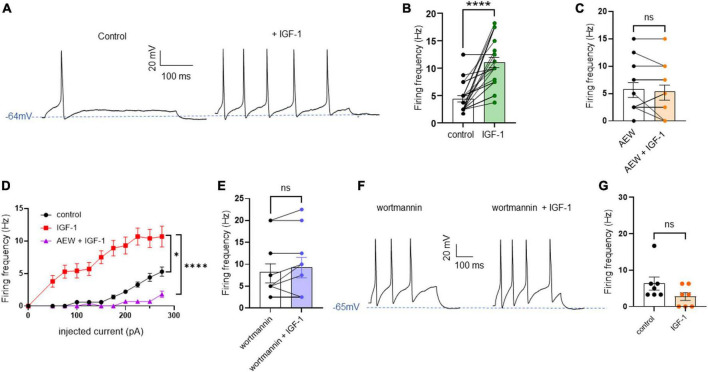
Acute exposure of DRG neurons to IGF-1 increases the spikes evoked by depolarizing current injection. **(A)** Representative traces of action potentials evoked by injecting 100 pA of depolarizing current in the same DRG neuron before and following 2 min exposure to 100 nM IGF-1. **(B)** Statistics of spike frequency evoked by the same depolarizing current injection (20–100 pA) in the same DRG neuron. The paired mean frequency was 4.4 ± 0.5 Hz and 11.1 ± 0.9 Hz before and after IGF-1 exposure, respectively; *n* = 25, two-tailed Wilcoxon matched-paired test, ****P < 0.0001. **(C)** The IGF-1-mediated increase in spike frequency was prevented by the pre-incubation with the IGF-1R antagonist NVP-AEW541 (2 μM, 1 h pre-incubation). Paired mean frequency was 5.8 ± 1.3 Hz and 5.4 ± 1.4 Hz for NVP-AEW541 alone and NVP-AEW541 + IGF-1, respectively; *n* = 12, two-tailed paired *t*-test, *t* = 0.6916, df = 11, *P* = 0.5035. **(D)** F-I curve showed that at all depolarizing current injections (from 0 to 275 pA in 25 pA increments) IGF-1 significantly increased the frequency of evoked spikes and that the IGF-1R antagonist NVP-AEW541 (2 μM) prevented the IGF-1 stimulating effect (*n* = 8; one-way ANOVA, Friedman test ****P < 0.0001; control vs. IGF-1, adjusted *P = 0.0417; control vs. AEW + IGF-1, adjusted *P* = 0.2209 and IGF-1 vs. AEW + IGF-1, adjusted ****P < 0.0001). **(E)** The IGF-1-mediated increase in spike frequency was prevented by the pre-incubation with the PI3K blocker wortmannin (0.2 μM, 1 h pre-incubation). Paired mean frequency was 8.3 ± 2.2 Hz and 9.3 ± 2.3 Hz for wortmannin alone and wortmannin+IGF-1, respectively; *n* = 10, two-tailed paired *t*-test, *t* = 1.500, df = 9, *P* = 0.1679. **(F)** Representatives traces of spikes evoked by injecting 100 pA of depolarizing current in the same DRG neuron, exposed to wortmannin alone and to wortmannin +IGF-1. **(G)** Statistics of the effects of IGF-1 on the intrinsic excitability of large-diameter (> 40 μm) non-nociceptive DRG neurons by injecting a ramp of depolarizing current (0–600 pA for 800 ms) in the same neuron. The paired mean frequency was 6.3 ± 2.1 Hz and 2.2 ± 1.0 Hz before and after IGF-1 exposure, respectively (*n* = 7, two-tailed Wilcoxon matched-pairs signed rank test, *P* = 0.2500).

**FIGURE 3:**
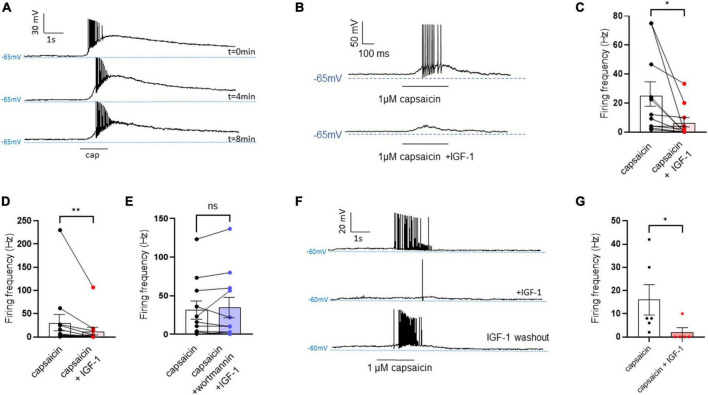
Acute exposure of DRG neurons to IGF-1 decreases the spikes evoked by capsaicin. **(A)** Representative traces of capaicin-induced spike burst. Capsaicin (1 μM) was applied for 1,500 ms every two minutes using a fast perfusion system to check the stability of the neuron’s response. Traces at 0, 4 and 8 min are shown. **(B)** Representative trace in the same DRG neuron of spikes evoked by capsaicin (1 μM) fast application for 500 ms without and with IGF-1 (100 nM). **(C)** Statistics of spike frequency evoked in the same neuron by fast application (0.5–3 s) of 1 μM capsaicin alone (25.1 ± 8.4 Hz) or capsaicin and acute 100 nM IGF-1 (6.3 ± 3.3 Hz), (*n* = 11, two-tailed paired *t*-test, *t* = 2.531, df = 10, *P = 0.0298). **(D)** Statistics of spike frequency evoked in the same neuron by fast application (1–3 s) of capsaicin alone (29.6 ± 17.3 Hz) or capsaicin with 2 min IGF-1 in the bath (11.4 ± 8.1 Hz), (*n* = 13, two-tailed Wilcoxon matched-paired test, **P = 0.0015). **(E)** Statistics of spike frequency evoked in the same neuron by fast application (1–3 s) of capsaicin alone (31.6 ± 11.7 Hz) or capsaicin+IGF-1+wortmannin (34.9 ± 13.2 Hz) (*n* = 11, two-tailed paired *t*-test, *t* = 0.8952, df = 10, *P* = 0.3917). **(F)** Representative traces of the washout effect after IGF-1 application on the same DRG neuron. Upper trace shows the spike burst evoked by 1 μM capsaicin for 2 s, the middle trace shows the effect of 2 min incubation with IGF-1 (100 nM) and 1 μM capsaicin application for 2 s on the same cell. Lower trace shows the 4 min washout of IGF-1 and 1 μM capsaicin application for 2 s on the same cell. **(G)** Statistics of the effects of IGF-1 on capsaicin-evoke spike burst in two independent groups of DRG neurons (mean spike frequency from 16.0 ± 6.5 Hz to 2.0 ± 2.0 Hz, *n* = 6, two-tailed Mann–Whitney test, *P = 0.0390).

**FIGURE 4:**
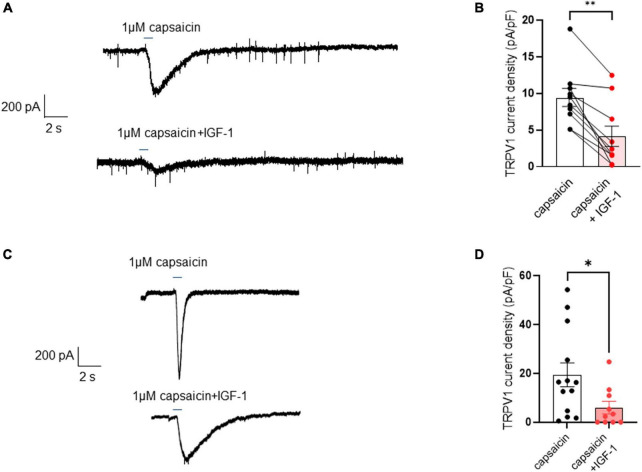
Acute exposure of DRG neurons to IGF-1 decreases the TRPV1 current evoked by capsaicin. **(A)** Representative trace of TRPV1 inward current evoked in the same DRG neuron by fast application (2.5 s) of 1 μM capsaicin without and with 100 nM IGF-1. **(B)** Statistics of TRPV1 current density induced by fast application (0.5–3 s) of 1 μM capsaicin (9.4 ± 1.2 pA/pF) or capsaicin and acute IGF-1 (4.1 ± 1.3 pA/pF), (*n* = 10, two-tailed Wilcoxon matched-paired test, **P = 0.002). **(C)** Representative trace of TRPV1 inward current evoked in one DRG neuron by fast application (0.5 s) of 1 μM capsaicin without and in another neuron with 100 nM IGF-1. **(D)** Statistics of TRPV1 current density induced by fast application (0.5 s) of 1 μM capsaicin in the absence or presence of 100 nM IGF-1 as determined in two independent groups of neurons (*n* = 13 and 10; from 19.4 ± 4.9 pA/pF to 6.0 ± 2.6 pA/pF, two-tailed Mann–Whitney test, *P = 0.0241).

**FIGURE 5:**
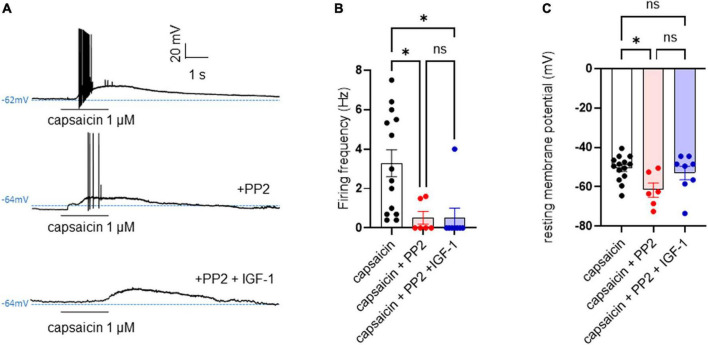
PP2 hyperpolarized the resting membrane potential, potently depressed the capsaicin-evoked spike burst but did not alter the IGF-1 inhibition of the hyperexcitability triggered by capsaicin. **(A)** Representative traces of membrane potentials of a DRG neuron upon application of 1 μM capsaicin for 2 s in the absence, presence of 10 μM PP2 and then 10 μM PP2+100 nM IGF-1. **(B)** Statistics of firing frequency triggered by capsaicin without and with 10 μM PP2 and 10 μM PP2+100 nM IGF-1. Mean spike frequencies of 3.3 ± 0.7 Hz, 0.5 ± 0.3 Hz and 0.5 ± 0.5 Hz for capsaicin, capsaicin+PP2 and capsaicin+PP2+IGF-1, respectively [one-way ANOVA *F*(2, 25) = 6.707 with Tukey’s multiple comparisons test: capsaicin vs. capsaicin + PP2, *P = 0.0239; capsaicin vs. capsaicin + PP2 +IGF-1, *P = 0.0118; capsaicin + PP2 vs. capsaicin + PP2 +IGF-1, *P* = 0.9999]. **(C)** Statistics of resting membrane potentials (RMP). RMPs of −50.5 ± 1.7 mV, −61.7 ± 3.5 mV and −53.0 ± 3.4 mV for capsaicin, capsaicin+PP2 and capsaicin+PP2+IGF-1, respectively; [one-way ANOVA *F*(2, 25) = 4.195 with Tukey’s multiple comparisons test: capsaicin vs. capsaicin + PP2, *P = 0.0209; capsaicin vs. capsaicin + PP2 + IGF-1, *P* = 0.7592; capsaicin + PP2 vs. capsaicin + PP2 + IGF-1, *P* = 0.1274].

First, we explored the effects of acute exposure of IGF-1 (100 nM) on the intrinsic excitability of cultured neonatal rat DRG neurons. For recording of nociceptive sensory neurons, only small-diameter (20–30 μM) DRG neurons were examined in the current-clamp mode of the whole-cell patch-clamp technique. Control DRG neurons did not exhibit spontaneous firing and presented a resting membrane potential (RMP) of −56.2 ± 1.8 mV (*n* = 18; Figure 1C). Figure 1A shows the recording of such a control neuron that did not display spontaneous firing but was excitable since it produced spikes upon a ramp of depolarizing current injection (from 0 to 600 pA for 800 ms). Within 1 min application of 100 nM IGF-1, the RMP of DRG neurons significantly depolarized to −43.5 ± 2.7 mV (Figure 1C; *n* = 18; two-tailed paired *t*-test, *t* = 6.136, df = 17; ****P < 0.0001). Moreover, out of 18 recorded neurons, 12 exhibited spontaneous firing (Figure 1B), which was prevented by the prior incubation with the IGF-1R antagonist NVP-AEW541 (2 μM, 1 h pre-incubation), suggesting that the effect produced by IGF-1 was mediated by the IGF-1R [Figure 1D; *n* = 18. Firing frequency (Hz) was 0, 0.094 and 0, in the absence, presence of IGF-1 and IGF-1+ NVP-AEW541, respectively; Kruskal–Wallis test, ****P < 0.0001 and ***P = 0.0002]. Then, for the DRG neurons that did not fire spontaneously upon IGF-1 exposure, we examined the effect of acute application of IGF-1 (100 nM) on the frequency of spikes evoked by depolarizing current injection. In this set of experiments, out of 48 small-diameter DRG neurons recorded, 45 cells (95%) were responsive to IGF-1 (100 nM) with an increased intrinsic excitability. This predominant sensitivity to the IGF-1 effect is in line with the abundant expression of the IGF-1R, previously shown in mouse DRG neurons (Lin et al., 2016a,b). Following depolarizing current injection, IGF-1 significantly increased the frequency of spikes (Figures 2A, B; *n* = 25. For 20–100 pA current injection, the paired mean frequency was 4.4 ± 0.5 Hz and 11.1 ± 0.9 Hz before and after IGF-1 exposure, respectively; two-tailed Wilcoxon matched-paired test, ****P < 0.0001). This IGF-1-mediated increase in spike frequency was prevented by the pre-incubation with the IGF-1R antagonist NVP-AEW541 (2 μM) (Figure 2C; *n* = 12, two-tailed paired *t*-test, *t* = 0.6916, df = 11, *P* = 0.5035). In addition, a full F-I curve showed that at all depolarizing current injections IGF-1 significantly increased the frequency of evoked spikes and that the IGF-1R antagonist NVP-AEW541 (2 μM) prevented the IGF-1 stimulating effect (Figure 2D; *n* = 8; one-way ANOVA, Friedman test ****P < 0.0001; control vs. IGF-1, adjusted *P = 0.0417; control vs. AEW + IGF-1, adjusted *P* = 0.2209 and IGF-1 vs. AEW + IGF-1, adjusted ****P < 0.0001). Interestingly, the IGF-1-mediated increase in spike frequency was precluded by the PI3Kinase inhibitor wortmannin (0.2 μM, 1 h pre-incubation) (Figures 2E, F; *n* = 10, two-tailed paired *t*-test, *t* = 1.500, df = 9, *P* = 0.1679). Altogether, these data indicate that acute IGF-1 exposure to small-diameter DRG neurons increased their intrinsic excitability, an effect mediated by the IGF-1R and involving the PI3Kinase pathway. For comparison, we checked the effects of IGF-1 on the intrinsic excitability of large-diameter (> 40 μm) non-nociceptive DRG neurons by injecting a ramp of depolarizing current (0–600 pA for 800 ms). Despite the significant expression of the IGF-1R in medium and large diameter DRG neurons (Lin et al., 2016a,b), results show that IGF-1 did not affect significantly the evoked spike frequency in these cells (Figure 2G; *n* = 7, two-tailed Wilcoxon matched-pairs signed rank test, *P* = 0.2500). Then, we examined whether acute IGF-1 could also affect the spikes triggered by TRPV1 channel activation with 1 μM capsaicin. However, high capsaicin concentrations are known to produce acute short-term desensitization, mainly arising from the large Ca^2+^ influx through the channel (Jung et al., 2004; Maximiano et al., 2024). Indeed, calmodulin binds to both the N- and C-terminal regions of the channel. Notably, Ca^2+^-Calmodulin binding to the N terminal region of the channel promotes channel desensitization (Lishko et al., 2007). Furthermore, it has been shown that divalent cations such as Ca^2+^ are chelated by the triphosphate portion of ATP, preventing its binding to the channel, which may also contribute to TRPV1 desensitization (Lishko et al., 2007). Therefore, to limit TRPV1 desensitization, the external solution did not include added Ca^2+^ and instead contained 2 mM EGTA, while the internal solution was titrated to 135 nM free Ca^2+^ (with EGTA). In this set of experiments, out of 117 small-diameter DRG neurons recorded, 88 (75%) were responsive to capsaicin application and among them, 100% responded to IGF-1. In fact, under these experimental conditions capsaicin triggered an inward TRPV1 current with Na^+^ as a charge carrier. Thus, Figure 3A shows that fast application of 1 μM capsaicin induced a burst of spikes, which remained stable without detectable desensitization, when capsaicin was applied to the same DRG neuron repeatedly every 2 min. Surprisingly, concomitant exposure of the same DRG neuron to 100 nM IGF-1 produced a profound inhibition of the spike firing evoked by capsaicin (Figure 3B). This major depression of spike firing was equally observed when IGF-1 was acutely applied either by the fast application system together with capsaicin for 0.5–3 sec (Figure 3C; *n* = 11, mean spike frequency from 25.1 ± 8.4 Hz to 6.3 ± 3.3 Hz, two-tailed paired *t*-test, *t* = 2.531, df = 10, *P = 0.0298) or in the bath perfusion for 2 min (Figure 3D; *n* = 13. mean spike frequency from 29.6 ± 17.3 Hz to 11.4 ± 8.1 Hz, two-tailed Wilcoxon matched-paired test, **P = 0.0015). Figure 3F shows that the depressing effect of IGF-1 is reversible and could be washed out. The IGF-1-mediated inhibition of the spike bursts triggered by capsaicin was prevented by wortmannin (Figure 3E; *n* = 11, two-tailed paired *t*-test, *t* = 0.8952, df = 10, *P* = 0.3917). To definitely exclude a potential bias of an overestimated IGF-1 effect due to a possible desensitization of the spike burst signal evoked by the repeated application of 1 μM capsaicin to the same DRG neuron, we measured the impact of IGF-1, not in a pairwise manner, but on a separate group of DRG neurons (Figure 3G). Similar to the experiments performed on the same neuron, IGF-1 exposure concomitant to capsaicin application, led to a profound depression of the spike bursts and this effect was reversible upon IGF-1 washout (Figures 3F, G; *n* = 6; mean spike frequency from 16.0 ± 6.5 Hz to 2.0 ± 2.0 Hz, two-tailed Mann–Whitney test, *P = 0.0390). To corroborate the depressing action of acute IGF-1 on the spike firing evoked by capsaicin, we checked the effects of acute IGF-1 on the inward TRPV1 current induced by capsaicin application under the voltage clamp configuration and using the fast perfusion system both in a pairwise fashion in the same DRG neuron but also in two independent groups of neurons. In the same DRG neuron, application of IGF-1 (100 nM) potently reduced the TRPV1 current density (Figures 4A, B; *n* = 10 from 9.4 ± 1.2 pA/pF to 4.1 ± 1.3 pA/pF; two-tailed Wilcoxon matched-paired test, **P = 0.002). Similar results were obtained when the experiments were performed in two independent groups of DRG neurons (Figures 4C, D). Under these conditions, IGF-1 significantly depressed the TRPV1 current density (Figure 4D; *n* = 13 and 10; from 19.4 ± 4.9 pA/pF to 6.0 ± 2.6 pA/pF, two-tailed Mann–Whitney test, *P = 0.0241). In an attempt to elucidate by which mechanisms acute IGF-1 reduces the spike burst evoked by capsaicin, we explored the possible involvement of the Src kinase, a member of the non-receptor tyrosine kinase family, using the specific c-Src kinase inhibitor PP2. Indeed, one of the IGF-1R signaling pathways (canonical) involves the activation of the PI3Kinase, which can activate the downstream Src kinase that binds to, phosphorylates and activates the TRPV1 channel (Jin et al., 2004; Zhang et al., 2005; Troncoso et al., 2014; Hakuno and Takahashi, 2018; Maximiano et al., 2024). While inhibition of the Src kinase by PP2 (10 μM) potently reduced the spike burst triggered by capsaicin, it did not alter the IGF-1 depression of capsaicin-evoked spikes [Figures 5A, B; *n* = 14, mean spike frequencies of 3.3 ± 0.7 Hz, 0.5 ± 0.3 Hz and 0.5 ± 0.5 Hz for capsaicin, capsaicin+PP2 and capsaicin+PP2+IGF-1, respectively; one-way ANOVA *F*(2, 25) = 6.707 with Tukey’s multiple comparisons test: capsaicin vs. capsaicin + PP2, *P = 0.0239; capsaicin vs. capsaicin + PP2 +IGF-1, *P = 0.0118; capsaicin + PP2 vs. capsaicin + PP2 +IGF-1, *P* = 0.9999]. Interestingly, treatment of DRG neurons with PP2 significantly hyperpolarized the resting membrane potential (RMP) [Figure 5C; *n* = 6–14, RMP of −50.5 ± 1.7 mV, −61.7 ± 3.5 mV and −53.0 ± 3.4 mV for capsaicin, capsaicin+PP2 and capsaicin+PP2+IGF-1, respectively; one-way ANOVA *F*(2, 25) = 4.195 with Tukey’s multiple comparisons test: capsaicin vs. capsaicin + PP2, *P = 0.0209; capsaicin vs. capsaicin + PP2 + IGF-1, *P* = 0.7592; capsaicin + PP2 vs. capsaicin + PP2 + IGF-1, *P* = 0.1274].

### Chronic IGF-1 treatment depressed the intrinsic excitability, upregulated the M-current density and markedly increased the spike firing evoked by capsaicin

**FIGURE 6:**
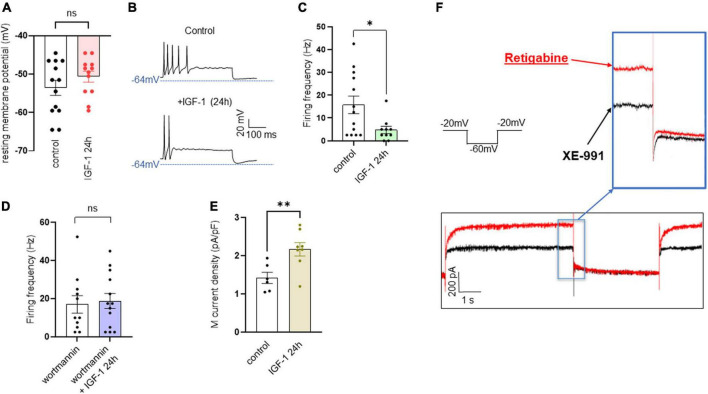
Chronic IGF-1 treatment decreases the spikes evoked by depolarizing current injection and increases the M-current density. **(A)** Following 24 h treatment with 100 nM IGF-1, the resting membrane potential remains similar to that of control neurons [**(A)** RMPs of −53.6 ± 2.0 mV (*n* = 13) and −50.7 ± 1.4 mV (*n* = 12) for control and IGF-1-treated neurons, respectively; two-tailed unpaired *t*-test, *t* = 1.178, df = 23, *P* = 0.2510]. **(B)** Representative traces of spikes evoked by injecting 250 pA of depolarizing current in a control DRG neuron (upper panel) and in a DRG neuron exposed to 100 nM IGF-1 for 24 h (lower panel). **(C)** Statistics of spike frequency evoked by the same depolarizing current injection (250 pA) in control neurons (15.8 ± 3.8 Hz, *n* = 13) and in neurons subjected to 24 h incubation with 100 nM IGF-1 (4.8 ± 1.6, Hz, *n* = 10); two-tailed unpaired Mann–Whitney test, *P = 0.0439. **(D)** The chronic depressing effect of IGF-1 on evoked spikes was prevented by prior incubation with wortmannin; after injection of 250 pA of depolarizing current, the spike frequency of wortmannin alone was 17.1 ± 4.6 Hz (*n* = 13) and of 24 h IGF-1+wortmannin was 18.9 ± 4.0 Hz (*n* = 11), two-tailed unpaired *t*-test, *t* = 0.2986, df = 22, *P* = 0.7681. **(E)** Statistics of M current density: control, 1.42 ± 0.14 pA/pF (*n* = 6) and 24 h IGF-1 2.17 ± 0.17 pA/pF (*n* = 8); two-tailed unpaired *t*-test, *t* = 3.128, df = 12, **P = 0.0087. **(F)** Representative trace of an M-current measured in a DRG neuron using the standard the tail protocol. While cells are held at −60 mV, a step to −20 mV is given for 6 s, to open the M-currents and remove residual inactivating voltage-dependent currents. Then, the voltage is brought back to −60 mV for 4 s to close M-currents, followed by another −20 mV step for 2 s. DRG neurons were first exposed to retigabine (10 μM), then incubated with XE991 (10 μM). The M-current is calculated by subtracting the peak tail current at −60 mV in the presence of RTG (red trace), to that in the presence of XE991 (black trace).

**FIGURE 7:**
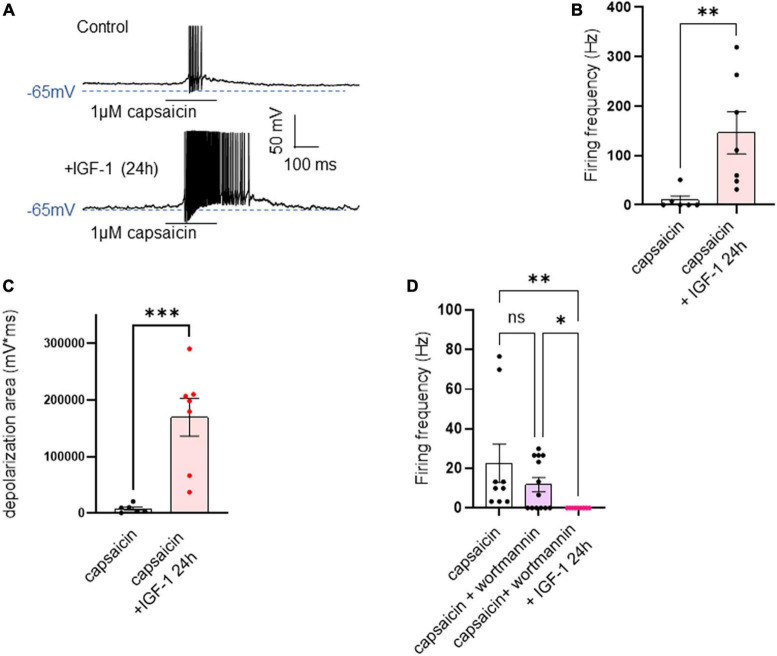
Chronic IGF-1 treatment markedly increased the spike firing evoked by capsaicin. **(A)** Representative traces of spikes evoked by the fast application (300 ms) of 1 μM capsaicin in a control DRG neuron (upper panel) and in a DRG neuron exposed to 100 nM IGF-1 for 24 h (lower panel). **(B)** Statistics of spike frequency evoked by 300 ms application of 1 μM capsaicin in control neurons (9.8 ± 8.3 Hz, *n* = 6) and in neurons treated by 100 nM IGF-1 for 24 h (145.8 ± 42.7 Hz, *n* = 7); two-tailed unpaired Mann–Whitney test, **P = 0.0047. **(C)** The depolarization area triggered by capsaicin was dramatically increased following chronic IGF-1 treatment [from 8,085 ± 2,949 mV*ms (*n* = 6) to 169,852 ± 33,403 mV*ms (*n* = 7); two-tailed unpaired *t*-test, *t* = 4.442, df = 11, ***P = 0.0010]. **(D)** This increase in capsaicin-induced spike bursting produced by chronic IGF-1 was prevented by pre-incubation with wortmannin and led to a complete shut-down of spiking [mean spike frequency: capsaicin 22.6 ± 9.7 Hz (*n* = 9), capsaicin+wortmannin 11.8 ± 3.6 Hz (*n* = 13) and 24 h IGF-1+wortmannin+capsaicin 0 ± 0 Hz (*n* = 9); Kruskal–Wallis test, significantly reduced **P = 0.0017]. Capsaicin + wortmannin vs. capsaicin + wortmannin + IGF-1 24 h. Significantly larger. Kruskal-Wallis test, *P = 0.0362.

In contrast to the acute IGF-1 exposure, chronic 24 h treatment of small-diameter DRG neurons with IGF-1 (100 nM) did not produce a depolarization of the resting membrane potential, which in fact remains similar to that of control neurons [Figure 6A; RMPs of −53.6 ± 2.0 mV (*n* = 13) and −50.7 ± 1.4 mV (*n* = 12) for control and IGF-1-treated neurons, respectively; two-tailed unpaired *t*-test, *t* = 1.178, df = 23, *P* = 0.2510]. Moreover, contrary to its acute effects, chronic (24 h) IGF-1 exposure, markedly reduced the spike frequency evoked by depolarizing current injection [Figures 6B, C; spike frequency after injection of 250 pA before 15.8 ± 3.8 Hz (*n* = 13) and after 24 h incubation with IGF-1 4.8 ± 1.6, Hz (*n* = 10); two-tailed unpaired Mann–Whitney test, *P = 0.0439]. Under these conditions, no significant differences were observed in the after-hyperpolarization (AHP) that follows the first spike after current injection [−1.6 ± 0.9 mV*ms (*n* = 11) and −0.9 ± 0.7 mV*ms (*n* = 11) for control and IGF-1-treated neurons, respectively; two-tailed Mann–Whitney test, *P* = 0.7243]. This chronic depressing effect of IGF-1 on evoked spikes was prevented by prior incubation with wortmannin [Figure 6D; spike frequency after injection of 250 pA wortmannin alone, 17.1 ± 4.6 Hz (*n* = 13) and 24 h IGF-1+wortmannin 18.9 ± 4.0 Hz (*n* = 11), two-tailed unpaired *t*-test, *t* = 0.2986, df = 22, *P* = 0.7681]. In this set of experiments, out of 49 neurons recorded, 47 (95%) were sensitive to the IGF-1 effects. As chronic IGF-1 treatment decreased DRG intrinsic excitability and knowing that IGF-1 is known to regulate various ion channels such as the A-type K^+^ channel (Xing et al., 2006; Wang et al., 2014) or voltage-gated Ca^2+^ channels (Blair and Marshall, 1997; Shan et al., 2003; Zhang et al., 2014), we examined the impact of chronic IGF-1 exposure on the M-type K^+^ current, which is a crucial player of neuronal excitability. M-currents were recorded using the classical tail protocol (Adams et al., 1982) (see “Materials and methods”) and was calculated by subtracting the peak tail current at −60 mV in the presence of RTG (10 μM), to that in the presence of XE991 (10 μM). The data show that 24 h IGF-1 (100 nM) treatment produced a 52% increase in M-current density [Figures 6E, F; control 1.42 ± 0.14 pA/pF (*n* = 6) and 24 h IGF-1 2.17 ± 0.17 pA/pF (*n* = 8), two-tailed unpaired *t*-test, *t* = 3.128, df = 12, **P = 0.0087]. Then, we examined whether chronic IGF-1 treatment affects the spikes triggered by TRPV1 channel activation. In this set of experiments, out of 59 neurons recorded, 44 (75%) were responding to the capsaicin application and among them 100% were sensitive to IGF-1 treatment. Figure 7 shows that 24 h exposure of DRG neurons to IGF-1 potently increased the spike burst evoked by capsaicin application [Figures 7A, B; mean spike frequency: capsaicin 9.8 ± 8.3 Hz (*n* = 6) and 24 h IGF-1+ capsaicin 145.8 ± 42.7 Hz (*n* = 7), two-tailed unpaired Mann–Whitney test, **P = 0.0047]. The whole depolarization area triggered by capsaicin was dramatically enhanced following chronic IGF-1 treatment [Figure 7C; from 8,085 ± 2,949 mV*ms (*n* = 6) to 169,852 ± 33,403 mV*ms (*n* = 7); two-tailed unpaired *t*-test, *t* = 4.442, df = 11, ***P = 0.0010]. Interestingly, the spikes evoked by capsaicin were accompanied by a much larger AHP in IGF-1-treated neurons as compared to control neurons [AHPs that follow the first spike evoked by capsaicin were −25 ± 2.5 mV*ms (*n* = 6) and −274 ± 75 mV*ms (*n* = 7) for control and IGF-1-treated neurons, respectively; two-tailed Mann–Whitney test, **P = 0.0012]. The potent boosting of capsaicin-induced spike bursting produced by chronic IGF-1 was prevented by pre-incubation with wortmannin and even led to a complete shut-down of spiking [Figure 7D; mean spike frequency: capsaicin 22.6 ± 9.7 Hz (*n* = 9), capsaicin+wortmannin 11.8 ± 3.6 Hz (*n* = 13) and 24 h IGF-1+wortmannin+capsaicin 0 ± 0 Hz (*n* = 9); Kruskal–Wallis test, significantly reduced **P = 0.0017].

## Discussion

A large panel of studies indicate that IGF1- and IGF-1R are expressed in small-diameter nociceptive DRGs and in spinal dorsal horn neurons (Craner et al., 2002; Hawkes and Kar, 2002; Dai et al., 2009; Takayama et al., 2011; Li et al., 2013; Wang et al., 2014; Zhang et al., 2014; Zhu et al., 2018; Tang et al., 2019; Ma et al., 2023). These neuronal cells convey pain signals from the peripheral to the central DRG terminals and project via the superficial layers of the spinal dorsal horn to higher brain centers of the CNS. DRG nociceptors express various receptors and ion channels that are critical players of neuronal excitability (Waxman and Zamponi, 2014; Ovsepian and Waxman, 2023). Among them, TRPV1, a ligand-gated nonselective cation channel, which triggers painful stimuli and the voltage-gated M-type K^+^ channel that dampens neuronal excitability (Barkai et al., 2017; Du et al., 2018; Rosenbaum et al., 2022). Many lines of evidence suggest that the IGF-1/IGF-IR signaling contributes to pain sensitivity, however, its potential modulation of TRPV1 and M-type K^+^ channel in DRGs remains unknown.

In this work, we show that acute IGF-1 exposure enhanced intrinsic DRG neuron excitability by depolarizing the resting membrane potential up to reaching threshold and leading to spontaneous firing in about two third of the recorded neurons (≈12 out of 18). For the neurons that did not fire spontaneously, acute IGF-1 perfusion significantly increased the frequency of spikes evoked by depolarizing current injection. The ligand concentrations known to activate the IGF-1 receptors usually range between 10 and 30 nM. Nevertheless, although we used a supraphysiological concentration of 100 nM, all acute IGF-1 actions on DRG nociceptors were prevented by the specific IGF-1R antagonist NVP-AEW541 suggesting that these effects are mediated by the IGF-1R. Interestingly, the IGF-1 actions on DRG neuron excitability were precluded by the PI3Kinase inhibitor wortmannin, suggesting an involvement of the PI3Kinase pathway. IGF-1R activates multiple pathways through its intrinsic tyrosine kinase activity and through coupling to heterotrimeric G protein (Troncoso et al., 2014). While a non-canonical IGF-1R signaling mechanism involving a pertussis-sensitive G_i/o_ protein has been described in various tissues (Troncoso et al., 2014; Zhang et al., 2014), two canonical IGF-1R signaling pathways are also known to operate: the phosphatidylinositol-3 kinase (PI3K)/Akt and the extracellular signal-regulated kinase (ERK) pathways (Troncoso et al., 2014; Hakuno and Takahashi, 2018). Many studies indicate that IGF-1 regulates various ion channels, notably voltage-gated K^+^ and Ca^2+^ channels. Indeed, IGF-1 was previously shown to increase T-type Ca^2+^ channel currents in small-diameter DRG neurons via IGF-1R and to increase action potential firing upon depolarizing current injection, in line with our results (Zhang et al., 2014). Furthermore, double staining in mouse DRGs indicated that IGF-1R was heavily colocalized with the T-type Cav3.2 channels (Zhang et al., 2014; Lin et al., 2016a,b). Importantly, IGF-1 was reported to increase the sensitivity of mice to both thermal and mechanical stimuli under conditions modeling chronic hind paw inflammation, and these effects were attenuated by intra-plantar injection of a T-type Ca^2+^ channel inhibitor (Zhang et al., 2014). However, an up-regulated activity of T-type Ca^2+^ channels following acute IGF-1 exposure cannot entirely account for the increased intrinsic DRG excitability we observed in our work. Indeed, the above-described IGF-1-mediated increase in T-type Ca^2+^ current was insensitive to the PI3Kinase pathway and was instead coupled to a pertussis-sensitive G protein–dependent PKCα pathway (Zhang et al., 2014). The IGF-1-triggered hyperexcitability that we observed in our study was clearly prevented by the PI3Kinase inhibitor wortmannin. Interestingly, a previous study performed in sensory trigeminal neurons, showed that acute IGF-1 treatment induces neuronal hyperexcitability via the inhibition of the A-type transient voltage-gated K^+^ current (Wang et al., 2014). It was shown that IGF-1 attenuates the I_A_ current through sequential activation of the PI3K- and c-Raf-dependent ERK1/2 signaling cascade. Wang et al. (2014) suggested that PI3K/c-Raf signaling may activate Map Kinase Kinase, which then phosphorylates ERK1/2 to inhibit the I_A_ current. In addition, a more recent work showed that cholecystokinin (CCK) binding to the CCK-type B receptor triggers a Gβγ-dependent PI3Kinase pathway, which activates the Src kinase that decreases the I_A_ current and as such enhances the firing rate of DRG neurons and the peripheral pain sensitivity in mice (Yu et al., 2019). Interestingly, a recent study showed that dopamine receptor R1 signaling in medium spiny neurons can increase intrinsic neuronal excitability by decreasing the M-type K^+^ current via ERK-mediated phosphorylation of the Kv7.2 subunit of the M-channel (Tsuboi et al., 2022). In this context, the intrinsic hyperexcitability we observed following acute IGF-1 application may likely arise from a decrease in I_A_ or/and M-currents via the PI3K-Src kinase and c-Raf-dependent ERK1/2 signaling pathway. This assumption is also in line with our data showing that application of the Src kinase inhibitor PP2 hyperpolarizes the resting membrane potential (Figure 5).

Our data indicate that IGF-1 exerted dual actions on DRG excitability. In contrast to the hyperexcitability induced on DRG intrinsic properties, acute IGF-1 inhibited the TRPV1 current, and the frequency of spikes evoked by capsaicin, an activity that was prevented by PI3Kinase blockade. While our data are in agreement with previous work showing that acute IGF-1 exposure increases DRG intrinsic excitability and neuronal firing (Zhang et al., 2014), our results are not consistent with a previous study showing that both insulin and IGF-I enhance TRPV1-mediated membrane currents in heterologous expression systems and cultured rat DRG neurons (Van Buren et al., 2005). Though the reason for this discrepancy is not clear, it is possible that distinct experimental conditions, notably the use of low pH to activate the TRPV1 channel may account for the differences. We found that the inhibition of the Src kinase by PP2 potently depressed the capsaicin-evoked spike burst but did not alter the IGF-1 inhibition of the spike bursts triggered by capsaicin. This result excludes for two reasons the direct involvement of Src kinase in the inhibitory effect of IGF-1 on capsaicin-evoked spike bursts: (a) the Src kinase inhibitor PP2 does not affect the IGF-1 depression of capsaicin-evoked spikes; (b) in principle, one of the IGF-1R signaling pathways (canonical) involves the activation of the PI3Kinase, which can activate the downstream Src kinase. Then, we should expect that IGF-1, like NGF, increases capsaicin-evoked spike burst, since Src kinase binds to, phosphorylates and activates the TRPV1 channel (Jin et al., 2004; Zhang et al., 2005; Troncoso et al., 2014; Hakuno and Takahashi, 2018; Maximiano et al., 2024). However, our current-clamp and voltage-clamp experiments clearly showed that IGF-1 has an inhibitory effect both in pairwise and independent measurements (see, Figures 3, 4). This result may be accounted for by two complementary and additive mechanisms: (i) Ca^2+^ influx through TRPV1 can activate Ca^2+^-sensitive phospholipase C isoforms (e.g., PLCδs) leading to the depletion of phosphatidylinositol 4,5 bisphosphate (PIP2), which in turn may decrease TRPV1 channel activity (Rohacs, 2015). Indeed, PIP2 is known to be a positive modulator of TRPV1 at strong stimulus strength by direct interaction with the channel (Rohacs, 2015; Gada and Logothetis, 2022). (ii) Activation of the IGF-1R through the canonical PI3Kinase pathway will phosphorylate PIP2 into PIP3, which in turn will activate Akt/PKB kinase via PDK1 (Hakuno and Takahashi, 2018). The resulting decrease in PIP2 levels to the benefit of PIP3 may additionally decrease the activity of the TRPV1 channel. Along this line, a previous work showed that in nociceptive DRG neurons, haploinsufficiency of type 1 phosphatidylinositol 4-phosphate 5-kinase (PI4,5K), the enzyme that generates PIP2 by phosphorylating the 5′ position on the inositol ring of phosphatidylinositol 4-phosphate, decreases the TRPV1 current activated by 1 μM capsaicin (Wright et al., 2014). Moreover, haploinsufficiency of PI4,5K reduces pro-nociceptive receptor signaling and TRPV1 sensitization in DRG neurons as well as thermal and mechanical hypersensitivity in mouse models of chronic pain (Wright et al., 2014).

Remarkably, chronic (24 h) IGF-1 treatment produced opposite effects to those obtained with acute application. Indeed, 24 h IGF-1 exposure depressed the DRG intrinsic excitability as reflected by a decrease in the frequency of spikes evoked by depolarizing current injection. In addition, we found that chronic IGF-1 significantly upregulated the M-current density, which may at least partially contribute for its depressive impact on DRG intrinsic firing, for the absence of depolarization of the resting membrane potential like that found upon acute IGF-1 exposure and for the large AHP that accompanies the spikes evoked by capsaicin. Indeed, the M-current together with the SK channels were shown to contribute to the medium AHP (Storm, 1989, 1990), while the BK channels are involved in the fast AHP and more recently, IK channels (SK4) were suggested to contribute to the slow AHP (Storm, 1990; Sahu and Turner, 2021). This long-term effect may reflect a homeostatic compensation process in front of the chronic IGF-1 perturbation. This intrinsic compensatory process may also include homeostatic changes in other ion channel activities. A previous study also revealed that chronic IGF-1 treatment of HEK293 cells can upregulate outwardly rectifying whole-cell K^+^ currents and the mRNAs encoding for Kv1.1, Kv1.2 and Kv1.3 channels and these effects were blocked by the PI3Kinase inhibitor wortmannin (Gamper et al., 2002). Besides the decreased intrinsic DRG excitability, chronic IGF-1 strikingly enhanced the excitability triggered by capsaicin-mediated TRPV1 activation. Interestingly, a previous work showed that bone cancer rats inoculated with rat mammary gland carcinoma cells, suffered from thermal hyperalgesia and mechanical allodynia (Li et al., 2014); they also exhibited increased IGF-1 expression that triggered in DRG nociceptors upregulation of TRPV1 current density and TRPV1 protein expression (Li et al., 2014). Thus, our *in vitro* data of chronic IGF-1 exposure may reflect the situation observed in chronic pain *in vivo* where IGF-1/IGF-1R signaling triggers increased excitability induced by TRPV1 activation. This plasticity of TRPV1 expression may be a more general feature of chronic pain signals since a recent study found that during colitis TRPV1 protein levels were significantly increased by nerve growth factor (NGF) in specific rat colonic afferent neurons of both L1 and S1 DRG neurons (Shen et al., 2017). This TRPV1 protein up-regulation in DRGs was modulated by activation of the PI3K/Akt pathway both *in vivo* and *in vitro* (Shen et al., 2017).

Altogether, our data suggest that *in vitro* IGF-1 exerts complex effects on DRG neuron excitability as revealed by its dual and opposite actions upon acute and chronic exposures. This feature probably reflects the complexity of IGF-1 action *in vivo*, where many studies showed that the peripheral IGF-1/IGF-1R pathway mostly plays a role in promoting pain, while the central (spinal cord) IGF-1/IGF-1R pathway mainly play a role in relieving pain (Ma et al., 2023). Regarding its complexity, the IGF-1/IGF-1R system may not be an optimal target for the clinical treatment of chronic pain. Across many drug development programs targeting either TRPV1, Nav1.7 or Kv7.2/3 channels, significant evidence has been accumulated to support these channel proteins still as highly relevant targets.

## Data availability statement

The raw data supporting the conclusions of this article will be made available by the authors, without undue reservation.

## Ethics statement

The animal study was approved by the Institutional Animal Care and Use Committee of Tel-Aviv University, Israel (authorization number 01-21-073). The study was conducted in accordance with the local legislation and institutional requirements.

## Author contributions

JP: Formal analysis, Investigation, Methodology, Writing – original draft. BA: Conceptualization, Funding acquisition, Methodology, Supervision, Validation, Writing – original draft, Writing – review & editing.
